# Optimization of lipid extraction from the oleaginous yeasts *Rhodotorula glutinis* and *Lipomyces kononenkoae*

**DOI:** 10.1186/s13568-018-0658-4

**Published:** 2018-08-06

**Authors:** Bruno Vasconcelos, José Carlos Teixeira, Giuliano Dragone, José António Teixeira

**Affiliations:** 1 Centre of Biological Engineering, University of Minho, Braga, Portugal; 2 Mechanical Engineering Department, University of Minho, Guimarães, Portugal; 3 Novo Nordisk Foundation Center for Biosustainability, Technical University of Denmark, Kongens Lyngby, Denmark

**Keywords:** Oleaginous yeasts, *Lipomyces kononenkoae*, Lipid extraction, *Rhodotorula glutinis*, Toluene, Cell rupture

## Abstract

The constant growing demand for vegetable oil for biodiesel and food is raising many environmental concerns about the sustainability of its production based on crops. Oleaginous yeasts show great potential to end with those concerns due to their high lipid productivity in small areas. To evaluate their productivity in lipids, an efficient and reproducible extraction process should be used. As no standard extraction process is available for the extraction of yeast lipids, an optimized extraction process is presented. In this work, the lipids extraction process for the yeasts *Rhodotorula glutinis* and *Lipomyces kononenkoae* is optimized using bead beating for cell rupture and introducing adaptations of the two most used extraction methods (Bligh and Dyer and Folch). For *Rhodotorula g.* the optimum extraction conditions are obtained by the Bligh and Dyer method applying 4.8 cycles of 47 s with 0.7 g of glass beads. For *Lipomyces k*. the optimum extraction conditions make use of the Folch method applying seven cycles of 42 s with 0.54 g of glass beads. These results reinforce the idea that, for each yeast, different extraction processes may be needed to correctly determine the lipid yield. The extraction procedure was further evaluated with less harmful solvents. Toluene was tested as a possible substitute of chloroform, and ethanol as a possible substitute of methanol. With the optimized extraction process, better results for *Lipomyces k*. were obtained using toluene and ethanol, while for *Rhodotorula g*. toluene proved to be a valid substitute of chloroform but ethanol is far less effective than methanol.

## Introduction

The growing demand for vegetable oils not only for food but also for the production of biodiesel has led to an increase in oil prices, despite the constant increase in its production, with serious environmental consequences related to deforestation for new plantations. As such, it has proved increasingly important to find solutions for sustainable production of vegetable oils or equivalents.

The oleaginous yeasts (OY) have been presented as a possible sustainable solution with great potential for the production of oils similar to vegetable oils, leading to a large number of studies aiming at the optimization of their lipid productivity; for this the most varied means and techniques have been proposed. OY can accumulate up to more than 70% lipids with a fatty acid profile similar to the vegetable oils (Ratledge 1991) with several advantages for lipid production over other sources, as they have rapid growth, require small areas for their cultivation and are much less affected by climatic conditions (Ageitos et al. 2011). However, with the current technology, biodiesel and vegetable oil equivalents production by oleaginous microorganisms (OM) is still not feasible because it is not cost-competitive (Probst et al. 2017; Whiffin et al. 2016) and thus further research needs to be done aiming to increase productivity and decrease production costs.

In order to correctly evaluate the productivity of the yeasts in lipids it is essential to use an extraction method as efficient and reproducible as possible. It would be important to have a standardized extraction method to accurately compare the results of different authors; however there is no extraction method equally efficient for different OM (Yeast, fungi, bacteria and microalgae) (Yu et al. 2015) and even studies with different species of microalgae or yeasts show that for different species the most effective method can be different (Lee et al. 2010; Prabakaran and Ravindran 2011; Bonturi et al. 2015). This is due to the fact that different species may present different physical properties such as different forms and different cell wall structure as well as different lipid compositions (Jacob 1992). Even in the same species of yeast there may be differences since the cell wall becomes thicker with growth and with the appearance of scars resulting from cell division (Jacob 1992). The development of thick cell walls makes them resistant to certain solvents (Ageitos et al. 2011). Effective cell disruption is thus a key step towards effective extraction in most OM. In order to cope with different lipid compositions it is generally advantageous to combine a polar and nonpolar solvent to extract a greater amount of lipids (Li et al. 2014). Combinations of Chloroform and Methanol are the most used in several methods of extraction and for the OY the most used methods are Bligh and Dyer (1959), Folch (1957) and adaptations/modifications of both.

In order to optimize OM cell disruption and consequently to improve extraction, several studies have been carried out using different methods of rupture (Schütte et al. 1983; Chisti and Moo-Young 1986; Cescut et al. 2011; Hegel et al. 2011; Prabakaran and Ravindran 2011; Jin et al. 2012; de Souza et al. 2014; Byreddy et al. 2015, 2016). The chemical rupture with HCl is one of the most used methods to extract lipids from OY and according to Yu et al. present even greater efficacy compared to other methods when applied to the yeast *Cryptococcus curvatus* (Yu et al. 2015); in this same study, bead-beating was shown to be an effective method of rupture for yeast and easy to scale-up with fairly close results to HCl digestion (Schütte et al. 1983). In addition, not using chemicals means less costs and absence of corrosion effects. As such, in this study, the cell rupture method was optimized in order to optimize the extraction of lipids from two OY (*Rhodotorula glutinis* and *Lipomyces kononenkoae*) applying adaptations of two of the extraction methods most frequently used in the literature (Folch and Bligh and Dyer). The selected method of cell rupture was mechanical rupture with glass beads using fast prep equipment (PBMS Cell disruptor Fast Prep-24, MP Biomedicals), which is recognized as a fairly effective method of cell rupture for yeast application. The yeasts used in this study are two OY with high potential for the production of lipids, one being the widely studied *Rhodotorula glutinis*, which can accumulate up to 72% lipids (Ratledge 1991; Ageitos et al. 2011) and the other is *Lipomyces kononenkoae* that also presents high potential as shown by Slininger et al. (2016). In addition, they present morphological differences and different cellular wall structure, which allows to evaluate the possible influence these properties have on the extraction efficiency.

Both Folch and Bligh and Dyer use chloroform and methanol in the extraction process, being chloroform highly dangerous and undesired in several solvent selection guides that take into account the impact on health and in the environment of various solvents (Alfonsi et al. 2008; Tobiszewski et al. 2015). As such it was decided to evaluate the possibility of using less hazardous solvents without losing extraction efficiency. Toluene and ethanol were the solvents chosen for testing. Although toluene is also considered problematic by some studies, it is a cheap solvent and several authors consider it an acceptable substitute for chloroform. Although dichloromethane is considered to be a better alternative to chloroform, it is also considered undesirable and toluene, a functional equivalent, is thus presented as a greener substitute in extraction processes (Alfonsi et al. 2008).

## Materials and methods

### Microorganisms, medium and chemicals

The oleaginous yeasts used in this study where *Lipomyces kononenkoae* PYCC 4052 and *Rhodotorula glutinis* PYCC 4177. All chemicals and reagents were of analytical grade. Yeast extract, peptone, dextrose, starch and glycerol were used for preparing the several media needed. Wastewater was obtained from the wastewater treatment plant of Frossos, Braga. Chloroform, methanol, ethanol and toluene, K_2_HPO_4_ and KCl where used in the extraction of the lipids. Fatty acid standards were used in the lipid analyses.

### Biomass cultivation

The yeasts were maintained in yeast extract peptone dextrose (YPD) plates (20 g/L glucose, 20 g/L peptone, 10 g/L yeast extract and 12.5 g/L agar).

The seed medium was YPD prepared with 20 g/L glucose, 20 g/L peptone, and 10 g/L yeast extract and the yeasts were cultured for 24 h at 28 °C and 180 rpm.

The culture medium used was urban wastewater supplemented with glycerol (5%) for *Rhodotorula glutinis* and starch (80 g/L) for *Lipomyces kononenkoae*. The choice of these media was due to the fact that in our laboratory we are evaluating the potential of these yeasts for the sustainable production of lipids for biodiesel. Yeasts were grown at 28 °C and 180 rpm for 7 days.

Cell mass growth was monitored by optical density at 600 nm.

After being cultivated for 7 days the yeasts were separated from the medium by centrifugation at 400 rpm (Scanspeed 416 benchtop centrifuge) and washed twice with distilled water. Subsequently they were lyophilized and stored at − 20 °C.

All media were sterilized at 121 °C for 15 min.

### Cell rupture

Cell rupture was performed using the Fast Prep equipment. Fast Prep equipment is a high-speed benchtop homogenizer able to simultaneous homogenize up to 24 samples in 2 mL tubes. The FastPrep instrument was developed for difficult and resistant samples and uses an optimized motion to disrupt cells through the multidirectional, simultaneous beating of specialized Lysing Matrix beads on the sample material. Shaking cycles of up to 60 s can be performed to rupture the cells at different speeds that can reach a maximum of 6.5 m/s. In this study, 2 mL screw cap tubes were used with glass beads of 425–600 μm (size recommended by the manufacturer for yeast cell rupture). The amount of biomass used was constant (0.05 g per tube) and the shaking velocity was also constant being maintained at maximum speed (6.5 m/s) on each cycle. The variables to analyze were the beads mass, number of cycles and the duration of each cycle. The beads weight to be evaluated was 0.2 g, 0.5 g and 0.8 g, the number of cycles 4, 6 and 8 and the time of each cycle 30 s, 45 s and 60 s.

The experimental design studying the effect of these variables on the efficiency of cell rupture was done using Statsoft’s Statistical Program 8.0 (Design of experiments).

### Extraction method

In the extraction process, 0.2 g of biomass was used in each experiment, being distributed in four tubes, dispensing 0.05 g of biomass in each together with the mass of beads established for each experiment. In each of the tubes, for the Bligh and Dyer extraction method, buffer and solvents are added together in the proportions described in the literature (0.3 mL of buffer, 0.35 mL of chloroform and 0.35 mL of methanol) while in the Folch method only solvents are added on the tubes (0.66 mL of chloroform and 0.33 mL of methanol).

A detailed description of the two extraction processes evaluated for the same cell rupture conditions is presented:

#### Bligh and Dyer (with modifications)

Many modifications of the Bligh and Dyer method are available, but we decided to adapt the method of Bligh and Dyer miniaturized used by Burja et al. (2007) that studied several methods for extracting fatty acids from *Thraustochytrium* sp. ONC-T18 (Burja et al. 2007). They obtained larger amounts of fatty acids using the method of Bligh and Dyer miniaturized that uses significantly less amount of solvents.

1. Immediately after cell rupture, the contents of the four tubes were transferred to a 25 mL falcon where chloroform, methanol and K_2_HPO_4_ buffer were added in the ratio of 1:1:0.9 to a final volume of 7.25 mL of each solvent and 6.525 mL of buffer.
2. After shaking the contents for 20 times, allow them to stand until phase separation.
3. Using calibrated micropipettes, recover 3/5 of the volume of chloroform from the lower layer, above the beads.
4. Place chloroform in a pre-weighed flask and evaporate.
5. For the evaporation is used a rotary evaporator. Set the bath temperature to 70 °C and the evaporation time to 30 min. After that, to make sure all is evaporated, transfer the flask to an oven at 105 °C for 1 h and after to a desiccator until the stabilization of the temperature. Weigh the flask.

The percentage of lipids (Lipid %) is determined gravimetrically:

$${\text{Lipid}}\;\% = \left( {{\text{TL}} \times {\text{TB}} \times 100 \times 5} \right)/3$$

where TL is the total extracted lipids and TB is the total biomass used.

#### Folch (with modifications)

Some adaptations where made to the Folch method in order to use the same total volume of solvents as in our adaptation of the Bligh and Dyer method.

1. Immediately after cell rupture, the contents of the four tubes were transferred to a 25 mL falcon where chloroform and methanol were added in the ratio of 2:1 to a final volume of 9.66 mL of chloroform and 4.83 mL of methanol. 2.9 mL of K_2_HPO_4_ buffer was further added.
2. Shake the contents 20 times and let stand until phase separation.
3. Recover 3/5 of the volume of chloroform from the lower layer, above the beads.
4. Place chloroform in a pre-weighed flask and evaporate.
5. After evaporation using a rotary evaporator, place the flask in an oven at 105 °C for one hour and after that place it in a desiccator; after stabilising of the temperature, weigh the flask.

The percentage of lipids (Lipid %) is determined gravimetrically:

$${\text{Lipid}}\;\% = \left( {{\text{TL}} \times {\text{TB}} \times 100 \times 5} \right)/3$$

where TL is the total extracted lipids and TB is the total biomass used.

### Lipid analyses

The extracted lipids were prepared for the determination of fatty acids contents, performing an acid transesterification. Heptadecanoic acid was used as internal standard. The methyl esters were analysed in a GC under the following conditions:

- Column TEKNOKROMA CN100 60 m × 0.25 mm 0.25 μm film thickness.
- Column temperature: 100 °C (5 min) heating ramp from 4 °C/min to 240 °C (5 min).
- Injector at 250 °C; split 1:100 injection volume: 1 μL of sample.
- Drag gas: He at 1 ml/min.
- Detector MS: temperature of ion trap 150 °C.
- Transfer line temperature: 200 °C.
- Manifold temperature: 50 °C.

Fatty acid peaks were identified according to a standard mixture (Supelco 37 component FAME mix, varied concentration in dichloromethane, Sigma) and retention times.

### Experimental design and optimization of lipid extraction yield by response surface methodology

A three-level-three-factor central composite rotatable design CCRD leading to 19 sets of experiments was employed to establish the influence on the lipid extraction yield of the variables mass of glass beads, number of cycles and time of each cycle. For statistical analysis, the independent variables were coded according to Eq. (1) where each independent variable is represented by xi (coded value), Xi (real value), X0 (real value at the center point) and ΔXi (step change value). The coding facilitated the computation for regression analysis.

1 $${\text{xi = }}\left( {{\text{Xi}} - {\text{X}}0} \right)/\Delta {\text{Xi}}$$

The range and levels of the variables are given in Table 1. The experiment in the central point was replicated five times to provide sufficient degrees of freedom for estimating the purely experimental uncertainty variance.

**Table 1:** Levels and range of the independent variables

Independent variable	Levels and range		
	− 1	0	1
Cycles	4	6	8
Time (s)	30	45	60
Beads (g)	0.2	0.5	0.8

The experimental results were fitted with a second-order polynomial equation by multiple regression analysis. The quadratic model for predicting the optimal point was expressed according to Eq. (2), where *ŷ*_*i*_ represents the response variable, *b*_0_ is the interception coefficient, *b*_*i*_, *b*_*ii*_ and *b*_*ij*_ are the regression coefficients, n is the number of studied variables, and *X*_*i*_ and *X*_*j*_ represent the independent variables. Where possible, the model was simplified by elimination of statistically insignificant terms.

2 $$\widehat{y}_{i} = b_{0} + \mathop \sum \limits_{i = 1}^{n} b_{i} X_{i} + \mathop \sum \limits_{i = 1}^{n} b_{ii} X_{i}^{2} \mathop \sum \limits_{i = 1}^{n - i} \mathop \sum \limits_{j = i + 1}^{n} b_{ii} X_{i} X_{j}$$

The quality of the fitted polynomial model was expressed by the coefficient of determination *R*^2^, and its statistical significance was checked by the *F*-test. The significance of the regression coefficients was tested by *t*-value.

Results were analyzed by the Experimental Design Module of the Statistica 8.0 software (Statsoft, USA). The model permitted evaluation of the effects of linear, quadratic and interactive terms of the independent variables on the chosen dependent variables.

## Results

To optimize the extraction process the Experimental Design Module of the Statistica 8.0 software (Statsoft, USA) was used. A three-level-three-factor central composite rotatable design CCRD lead to 19 sets of experiments for each yeast and extraction method. The efficiency of the extraction process was determined considering the lipid extraction yield as previously defined.

### Optimization of the extraction process for *Rhodotorula glutinis* and *Lipomyces kononenkoae*

The lipid extraction yield in each experiment for both yeasts and extraction methods is present in Table 2, and it can be observed that the maximum lipid extraction yield for *Rhodotorula g*. was the same for both Bligh and Dyer and Folch methods (23.5%), Table 2, although different optimal were obtained by the application of the Statistica 8.0 software (Table 3). For the Folch method applied to *Rhodotorula g*., the critical value of glass beads is higher then the observed maximum. That would point for the need of a new experimental design, but higher mass of glass beads brings technical problems. Moreover, it was experimentally observed that a higher mass of beads didn’t improve the lipid yield. Bligh and Dyer method is as efficient as Folch in the extraction process for *Rhodotorula*, with the advantage of needing less glass beads, requiring less time and posing less technical problems. Therefore, the estimated optimum extraction conditions for *Rhodotorula g*. are Bligh and Dyer method (adapted) applying 4.8 cycles of 47 s with 0.7 g of glass beads in each tube.

**Table 2:** Lipid extraction yield for each experiment for *Rhodotorula glutinis* and *Lipomyces kononenkoae* using Bligh and Dyer and Folch extraction methods

Experiment	Cycles	Time (s)	Glass beads (g)	*Rhodotorula glutinis*		*Lipomyces kononenkoae*	
				Bligh and Dyer (% Lipids)	Folch (% Lipids)	Bligh and Dyer (% Lipids)	Folch (% Lipids)
5	8	30	0.2	19.5	16.6	24.4	32.2
16 (C)	6	45	0.5	20.4	20.3	30.4	34.4
10	8	45	0.5	*23.5*	23.1	36.3	34.2
2	4	30	0.8	20.9	20.6	22.2	33.2
12	6	60	0.5	21.1	18.6	37.0	*35.8*
9	4	45	0.5	20.4	19.1	25.1	32.8
19 (C)	6	45	0.5	20.8	19.5	30.1	35.0
18 (C)	6	45	0.5	20.3	21.3	30.1	34.6
17 (C)	6	45	0.5	21.6	21.1	30.5	34.4
14	6	45	0.8	21.3	20.3	34.4	32.5
4	4	60	0.8	21.2	*23.5*	30.9	34.4
6	8	30	0.8	20.8	21.1	33.8	34.2
8	8	60	0.8	20.5	20.5	*37.8*	34.8
1	4	30	0.2	17.5	14.5	14.8	29.8
7	8	60	0.2	19.3	17.7	36.3	35.7
11	6	30	0.5	21.2	18.8	28.3	32.5
13	6	45	0.2	20.3	18.4	23.9	32.8
3	4	60	0.2	18.5	16.5	22.8	31.3
15 (C)	6	45	0.5	21.8	20.0	29.0	35.3

**Table 3:** Estimated optimum extraction conditions for the yeast *Rhodotorula glutinis* using the Bligh and Dyer and Folch methods

Method Factor	*Rhodotorula glutinis*					
	Bligh and Dyer			Folch		
	Observed minimum	Critical values	Observed maximum	Observed minimum	Critical values	Observed maximum
Cycles	4	*4.8*	8	4	6.5	8
Time (s)	30	*47*	60	30	46	60
Glass beads (g)	0.2	*0.7*	0.8	0.2	0.86	0.8

For *Lipomyces kononekoae*, the extraction using the Bligh and Dyer method presented some technical problems that forced us to exclude this method. Although the maximum yield using this method was a bit higher than using the Folch method, no reliable and reproducible results were obtained, this being related with the difficulty in keeping the layer of cells compact during the process of collecting the lower chloroform layer. After centrifugation three layers are obtained, the upper layer with the methanol, buffer and impurities, a lower layer with the lipids, chloroform and glass beads and a third layer in the middle formed by the cells. While with *Rhodotorula g*. that cell layer is stable and easy to remove, or pierce to reach the lower layer, in the case of *Lipomyces k*. the layer of cells is not stable and easily part of it moves into the lower layer. Since with the Folch method the chloroform layer is much bigger and 3/5 of that layer are recovered, the cells that mix with the lower layer end up not being collected like it happens with Bligh and Dyer. The inclusion of an extra extraction step, like filtrating the lower layer, would turn the process more complex and time consuming and that is not justifiable when similar yields are obtained, as is the case. Therefore, the optimum extraction conditions estimated for *Lipomyces k*. are Folch method (adapted) applying seven cycles of 42 s with 0.54 g of glass beads in each tube (Table 4).

**Table 4:** Estimated optimum extraction conditions for the yeast *Lipomyces kononenkoae* using the Bligh and Dyer and Folch methods

Method Factor	*Lipomyces kononenkoae*					
	Bligh and Dyer			Folch		
	Observed minimum	Critical values	Observed maximum	Observed minimum	Critical values	Observed maximum
Cycles	4	11	8	4	*7*	8
Time (s)	30	19	60	30	*42*	60
Glass beads (g)	0.2	0.72	0.8	0.2	*0.54*	0.8

The R^2^ obtained for *Lipomyces k*. using Folch method and *Rhodotorula g*. using Bligh and Dyer method was 0.896 and 0.759, respectively, which means the model for *Lipomyces k*. explains approximately 90% of the variability observed in the response and the model for *Rhodotorula g*. explains approximately 76%.

The optimum extraction conditions where experimentally verified for both yeasts and it was obtained a percentage of lipids of 24.6 ± 1.041 and 36.1 ± 0.737 for *Rhodotorula g*. and *Lipomyces k*., respectively, which validates the model.

A statistical analysis was carried out to identify the variables that had the greatest influence in the selected extraction processes for each yeast. Tables 5 and 6 show the Student’s *t* test and p-values used to determine the statistical significance of the independent variables [cycles, time (s) and beads (g)] on the response variable [extraction lipid yield (%)]. For *Lipomyces k*. all individual variables had significant (at 95% confidence level) effect on lipid extraction yield. The interaction effects where all non significant except for the interaction between cycles and beads. For *Rhodotorula g*. the only variable with significant (at 95% confidence level) effect was glass beads mass.

**Table 5:** Effect estimates, standard errors, t-test and p-values for the optimization of lipid extraction yield from *Lipomyces K*. according to the full factorial CCRD

Variables and interactions	Estimated effects	Standard errors	*t*-value	*p*-value
Cycles (L)	1.760	0.463	3.802	0.004
Cycles (Q)	− 2.012	0.886	− 2.272	0.049
Time (L)	1.200	0.463	2.592	0.029
Time (Q)	2.587	0.886	2.922	0.017
Beads (L)	1.300	0.463	2.808	0.020
Beads (Q)	− 3.712	0.886	− 4.192	0.002
1L by 2L	0.150	0.518	0.290	0.779
1L by 3L	− 1.550	0.518	− 2.995	0.015
2L by 3L	− 1.000	0.518	− 1.932	0.085

*L* linear, *Q* quadratic

**Table 6:** Effects estimates, standard errors, t-test and p-values for the optimization of lipid extraction yield from *Rhodotorula g*. according to the full factorial CCRD

Variables and interactions	Estimated effects	Standard errors	*t*-value	*p*-value
Cycles (L)	1.020	0.563	1.811	0.104
Cycles (Q)	0.333	1.077	0.309	0.764
Time (L)	0.140	0.563	0.249	0.809
Time (Q)	− 1.267	1.077	− 1.176	0.270
Beads (L)	1.920	0.563	3.410	0.008
Beads (Q)	− 1.967	1.077	− 1.826	0.101
1L by 2L	− 0.450	0.630	− 0.715	0.493
1L by 3L	− 0.900	0.630	− 1.430	0.187
2L by 3L	− 0.200	0.630	− 0.318	0.758

*L* linear, *Q* quadratic

### Lipid profile of *Rhodotorula glutinis* and *Lipomyces Kononenkoae*

The lipids obtained after extraction in the optimum conditions were analysed to determine the lipid profile. The relative mass percentage of the different fatty acids for each yeast is presented in Table 7.

**Table 7:** Relative mass percentage of fatty acid present in the lipid extract of *Rhodoturula glutini*s and *Lipomyces kononenkoae* after extraction using the optimum conditions of extraction

Fatty acid	C16	C16:1	C18:0	C18:1	C18:2
*Rhodotorula glutinis* (%)	22	–	5	66	6
*Lipomyces kononenkoae* (%)	32	5	6	57	–

### Extraction with less harmful solvents in the optimal conditions

As said before, more environmental friendly solvents are required on the development of lipids extraction processes and, in this regard, toluene and ethanol are potential substitutes for the substitution of chloroform and methanol, respectively. Taking this in consideration, new experiments for lipid extraction were done using new biomass of *Rhodotorula g*. and *Lipomyces k*. Moreover different combinations of these solvents were evaluated and the results are presented in Table 8.

**Table 8:** Percentage of lipids obtained extracting in optimum conditions for both yeasts using different combinations of solvents, and the percentual increase of the extraction in comparison with chloroform/methanol extraction

Solvents	Percentage of lipids (%)			
	*Rhodotorula glutinis*	Percentual increase	*Lipomyces kononenkoae*	Percentual increase
Toluene/methanol	17.0 ± 0.4	− 5.03	39.7 ± 2.2	15.7
Chloroform/ethanol	11.1 ± 0.6	− 37.99	29.5 ± 1.8	− 13.99
Toluene/ethanol	10.5 ± 0.4	− 41.34	39.4 ± 1.3	14.87
Chloroform/methanol	17.9 ± 0.1	0	34.3 ± 0.8	0

Extractions performed in triplicate

For *Rhodotorula g*., similar results were obtained using toluene instead of chloroform, confirming the possibility of replacing chloroform by toluene, a less harmful solvent. The substitution of methanol by ethanol demonstrated that this solvent is far less effective then methanol.

For *Lipomyces k*. toluene proved to be even more effective then chloroform and even combined with ethanol the results were similar to the combination toluene/methanol, proving that a combination of toluene/ethanol can be used as a less harmful extraction solvent.

## Discussion

For the two yeasts it was obtained two different optimum extraction processes, reinforcing the idea that, for each yeast, different extraction processes are needed to correctly determine the lipid yield. Bonturi et al. (2015) have determined that acid and enzymatic pre-treatment only brings significant improvement in the lipid extraction yield of both *Rhodosporidium toruloides* and *Lipomyces starkeyi* using Bligh and Dyer or Pederson methods and no significant improvement occurs when using Folch method. In our study, the importance of the pre-treatment with bead-beating for lipids extraction using both methods for two yeast strains is clearly shown. Once again, that reinforces the idea that for each yeast, different extraction processes may be needed. Different species may have different forms and different cell wall structure as well as different lipid compositions and may develop thicker cell walls depending on the time and growth conditions (Jacob 1992). The development of thick cell walls makes them resistant to certain solvents (Ageitos et al. 2011) and that implies that an effective cell disruption is thus a key step to assure an effective lipid extraction.

The lipid profile obtained for *Lipomyces kononenkoae* is in accordance with the results of Lategan (2017) and Hossack (1978) that also report that oleic acid is the dominant fatty acid, with a slightly higher concentration than the one obtained in this study, while the percentage of the palmitic, palmitoleic and stearic acid are similar (33.7%; 4%; and 6.6%, respectively). The lipid profile obtained for *Rhodotorula glutinis* is also in accordance with the literature, since Kot and Kurcz (2016) describe that the percentage of oleic acid in the total pool of fatty acids may exceed above 60%, the linoleic acid percentage ranges from above 5 to 25% and palmitic acid constitutes on average 10–30%. Fatty acid profiles have been shown to be quite consistent within a species if grown under consistent conditions (Sitepu et al. 2013), but some differences are expected due to the different growth conditions (Sargeant et al. 2014; Easterling et al. 2009).

In this study, it was also possible to enhance lipids extraction from *Lipomyces k*. using less harmful solvents as is the case with toluene and ethanol. For *Rhodotorula g*. toluene proved to be a valid substitute of chloroform but ethanol is far less effective for the extraction process then methanol. Methanol in general is more effective for the extraction than ethanol because of its higher polarity as co-solvents are used to break the connections of the polar lipids to the biomass (Dong et al. 2016). The higher effectiveness of methanol was more visible for *Rhodotorula g*. then for *Lipomyces k*. not only because *Lipomyces k*. had higher amount of storage neutral lipids but also probably due to the different morphologies they have. *Lipomyces k*. is a spherical cell with a single lipid droplet inside, while *Rhodotorula g*. has an elongated form, with multiple small lipid bodies. The cell rupture of a cell like *Lipomyces k*. might expose more the lipids for extraction then the rupture of *Rhodotorula glutinis*. Lipid droplets appear to bind to several organelles (Gao and Goodman 2015) which might imply that multiple droplets will have a higher number of connections than just one big droplet making harder to separate the lipids from the biomass. That is why a more polar co-solvent is more important for the extraction in *Rhodotorula g*. then in *Lipomyces k*. The higher effectiveness of Toluene over Chloroform for the extraction of lipids from *Lipomyces kononenkoae* may be related with the lower polarity of Toluene compared to Chloroform. Since “like dissolves like” is a basic principle in the solvent extraction procedures, neutral lipids will favorably interact with the relatively non-polar solvent molecules (Cooney et al. 2017) and in this case, lower polarity of the toluene is favorable for the extraction. In a study about the effect of the solvent polarity on the sesame seeds oil composition, Tir et al. observed that sesame oil was better extracted with less polar solvents although membrane lipids require polar solvents for its extraction (Tir et al. 2012). Murali et al. studied polar and nonpolar lipids and their fatty acid composition of a few *Fusarium* species (Murali et al. 1993) and observed that nonpolar lipids were more saturated than polar lipids. That helps to justify the better results of toluene with *Lipomyces k*. since this yeast has a higher amount of saturated lipids then *Rhodotorula g*.

As a result of this study, it was developed an optimized, new lipids extraction process specifically for each of the two yeasts studied. For *Rhodotorula g*. the optimum extraction conditions were obtained using the Bligh and Dyer method (adapted) applying 4.8 cycles of 47 s with 0.7 g of glass beads in each tube. For *Lipomyces k*. the optimum extraction conditions were obtained with the Folch method (adapted) applying seven cycles of 42 s with 0.54 g of glass beads in each tube.

## Abbreviations

OM: oleaginous microorganisms
OY: oleaginous yeasts
CCRD: central composite rotatable design
YPD: extract peptone dextrose
